# Bloodstain Pattern Analysis: implementation of a fluid dynamic model for position determination of victims

**DOI:** 10.1038/srep11461

**Published:** 2015-06-22

**Authors:** Nick Laan, Karla G. de Bruin, Denise Slenter, Julie Wilhelm, Mark Jermy, Daniel Bonn

**Affiliations:** 1 WZI, IoP, University of Amsterdam, Science Park 904, Amsterdam, Netherlands; 2 Netherlands Forensic Institute, Laan van Ypenburg 6, The Hague, Netherlands; 3 Mechanical Engineering, University of Canterbury, Private Bag 4800, Christchurch 8140, New Zealand

## Abstract

Bloodstain Pattern Analysis is a forensic discipline in which, among others, the position of victims can be determined at crime scenes on which blood has been shed. To determine where the blood source was investigators use a straight-line approximation for the trajectory, ignoring effects of gravity and drag and thus overestimating the height of the source. We determined how accurately the location of the origin can be estimated when including gravity and drag into the trajectory reconstruction. We created eight bloodstain patterns at one meter distance from the wall. The origin’s location was determined for each pattern with: the straight-line approximation, our method including gravity, and our method including both gravity and drag. The latter two methods require the volume and impact velocity of each bloodstain, which we are able to determine with a 3D scanner and advanced fluid dynamics, respectively. We conclude that by including gravity and drag in the trajectory calculation, the origin’s location can be determined roughly four times more accurately than with the straight-line approximation. Our study enables investigators to determine if the victim was sitting or standing, or it might be possible to connect wounds on the body to specific patterns, which is important for crime scene reconstruction.

Bloodstain Pattern Analysis (BPA) is defined as the study of the shapes, sizes, distribution and locations of bloodstains in order to determine the physical events which gave rise to their origin. For example, if an object (e.g., a hammer) strikes a volume of liquid blood (e.g., a victim), droplets diverge away from the origin through the air (see supplementary video) and when hitting a surface an impact pattern (Fig. 1a) will be formed^1^. In contrast to DNA analysis, which gives information about the donor of the blood (individualization), BPA may provide information about the events that have taken place during the crime. Among others, the investigator wants to know where the location of the blood source (also known as the ‘region of origin’), was during the blood shedding event. This information may substantiate or refute claims of, e.g., self-defense, which is very important in the court of law to avoid miscarriages of justice. Several methods exist to determine the region of origin, for example, the stringing method^2^, the tangent method^1^, or the mathematical method from Varney *et al.*^3^. Most methods assume the trajectories of the droplets to be a straight line instead of curved, neglecting gravity and air resistance (drag). This assumption causes an overestimation in height, which can be as large as 45 cm depending on the distance between origin and wall^4^. In order to take the effects of gravity and drag into account, the impact velocity of the blood droplet at the time it hits the surface is required^5^. Studies concerning impact of blood droplets indicate that it is possible to determine the impact velocity from the resulting bloodstain, but it is dependent on the volume of the stain which they could not directly measure^5^,^6^,^7^. These studies try to circumvent this problem by taking the number of spines, created around the stain, into account. However, recently we showed that it is possible to determine the volume of a dried bloodstain and that it is possible to calculate the volume of the original droplet prior to impact^8^. Accordingly, we developed a method which can be used to determine the impact velocity based on the width of an elliptical bloodstain and its original volume^9^. In this study, we show for the first time that it is possible to determine the volume of bloodstains from a real impact pattern and infer the impact velocity of each stain chosen by the expert. More importantly, we show that with this method we are able to take both gravity and drag into account when determining the trajectory of a blood droplet, and determine the region of origin of an impact pattern much more precise and accurate than the methods currently in use. This research enables the investigator to determine the location of the blood source in the room, and connect it to the position of the victim (like standing or sitting), or connect specific wounds to certain patterns. To determine the region of origin by taking gravity and drag into account, we require five parameters of each bloodstain: 1) location of the bloodstain in x, y, and z coordinates, 2) directional angle *γ*, 3) impact angle *α*, 4) volume of the original blood droplet, and 5) impact velocity of the blood droplet. The first parameter is trivial and measured easily, which is done for the current methods in use. The directional angle *γ* is measured by comparing the direction of travel to the vertical (Fig. 1b,c). The impact angle *α* can be determined from the shape of the stain as the width *W*_*max*_ and length *L*_*max*_ of the elliptical outline of the stain (Fig. 1c) are empirically related to the impact angle by *sinα* = *W*_*max*_/*L*_*max*_^10^. It is possible to determine the volume of a bloodstain^8^, however this has never been done before with bloodstains of an impact pattern. We will show that by means of a 3D surface scanner we can determine the volume of small (≈1 *μ*l) bloodstains in a non-intrusive and objective manner (see supplementary materials). For the final parameter, the impact velocity, recent studies for simple fluids suggest that it can be inferred from the maximum diameter that an impacting drop of known volume attains^5^,^9^,^10^,^11^,^12^,^13^. During impact upon a surface, droplets spread in a circular fashion, where spreading is driven by inertial forces and countered by capillary and viscous forces^9^,^14^,^15^. These forces can be quantified in terms of the Weber number, *We* = *ρD*_0_*v*^2^/*σ*, the ratio between the inertial and capillary force, and the Reynolds number *Re* = *ρD*_0_*v*/*η*, the ratio between the inertial and viscous forces. Here *ρ* denotes the density of the fluid, *D*_0_ the diameter of the droplet in flight, *v* the impact velocity of the droplet, *σ* the surface tension and *η* the viscosity of the fluid. As spreading slows down, the spreading droplet reaches its maximum diameter, after which it can either retract due to the capillary forces^15^ or remain pinned to the surface^16^, which is the case for blood droplets^17^. Recently, Laan *et al.*^9^ showed that spreading of droplets in general, including blood droplets, can be described by means of an interpolation between the spreading behavior for the capillary regime (*We*^1/2^)^11^,^17^ and the viscous regime (*Re*^1/5^)^10^,^18^. This interpolation resulted in the following relation:




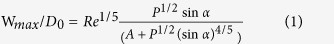




where the impact number *P* = *WeRe*^−2/5^ and *A* = 1.24 is a fitting parameter. By measuring the width, the impact angle and the volume of a bloodstain (*D*_0_), equation (1) can be solved numerically for the impact velocity. Accordingly, it is possible to solve the equations of motion for a projected droplet flying through the air, which is influenced by gravity and drag^19^ (see supplementary materials).

## Results

### Volume estimation of bloodstains

How much a droplet spreads is dependent on the volume, which can simply be deduced from the fact that bigger droplets create bigger stains. Accordingly, to determine the velocity of the droplet hitting the surface we require the original droplet volume. After a bloodstain dries, it leaves a residue, the dried red blood cells, the amount of which is dependent on the hematocrit value^8^. Plasma consists approximately out of 91% water and red blood cells contain approximately 70% water^20^. If we assume all water evaporates during the drying process, then the drying ratio κ should increase linearly as a function of the hematocrit value. Based on the volume ratio between dried and fresh blood it is possible to deduce the original volume of a bloodstain by measuring the volume of the dried stain. To do so, we use a 3D surface scanner (see supplementary materials). Under laboratory conditions, we measured in total the volume of 40 droplets.

**Figure 2:**
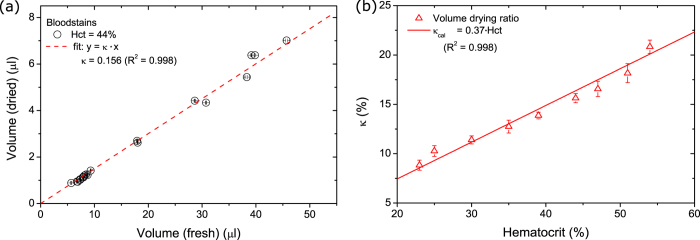
Volume and drying ratio measurements of bloodstains. (**a**) The volume of dried bloodstains obtained with the AreaScan3D plotted as a function of the volume of the fresh droplet determined by means of the weight and density. The fit to the data points gives us the calibrated volume ratio between the dried and fresh stains, which in this case (Hct = 44%) equals 15%. (**b**) The drying ratio κ as a function of the hematocrit value. The red line is the fit to the data points of which the slope is the drying ratio.

By plotting the dried volume (AreaScan3D) as a function of the fresh volume, obtained by means of weighing the fresh droplet, we are able to determine the drying ratio which is the slope of the fit to the data points (Fig. 2a). In our experiments the hematocrit value (Hct = 44%) was kept constant resulting in a drying ratio of 15.6%. In contrast, assuming all water evaporates, the drying ratio for Hct = 44% should be 18.2%. However, the data points shown in Fig. 2a, show a linear trend, so there seems to be a constant ratio between the fresh and dried blood volume values. We conclude that the measured dried bloodstain volume is dependent on the device used (see supplementary methods). We, therefore, determined a calibration curve such that the correct volume of the bloodstains can be estimated. The calibrated drying ratio (κ_*cal*_) enables us to determine what the original droplet volume was from a 3D scan of the dried bloodstain. Of course the hematocrit value can vary between individuals, and accordingly the drying ratio of the blood. Therefore, the measurements of Fig. 2a were repeated for blood with varying hematocrit values (Fig. 2b). As the hematocrit value increases, the drying ratio increases as well. By fitting a straight line to the data points, a relation is obtained to correlate the Hct value to κ:




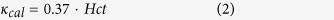




Eq. (2) enables us to determine what the drying ratio is for varying Hct values, i.e., different people, when using the AreaScan3D.

### Impact pattern analysis

As shown above, it is possible to determine every parameter necessary for the reconstruction of a trajectory including the effects of gravity and drag. Accordingly, we tested our method on stains of a real impact pattern at the Netherlands Forensic Institute (NFI) where we created eight impact patterns (n = 8) from a distance of one meter. We show how accurate and precise we can determine the location of the origin by determining the curved trajectory of each bloodstain chosen by a BPA expert.

**Figure 3:**
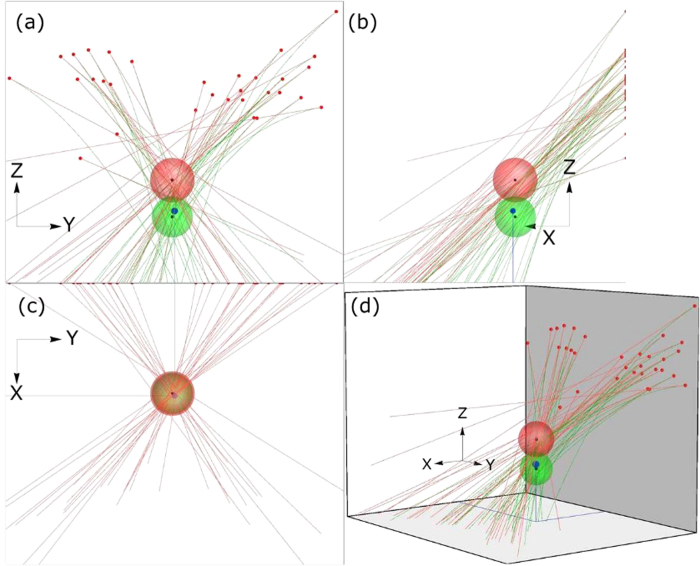
Example of the region of origin visualization of an impact pattern where red is the straight-line approximation and green is our gravity included method. (**a**) Front view, (**b**) side view, (**c**) top view, (**d**) 3D view. The red dots represent the bloodstains chosen for analysis. The red lines and green curves represent the trajectories determined with the straight-line approximation and gravity included method, respectively. The blue dot depicts the true origin. The red and green spheres represent the *RO* of the two methods.

**Figure 4:**
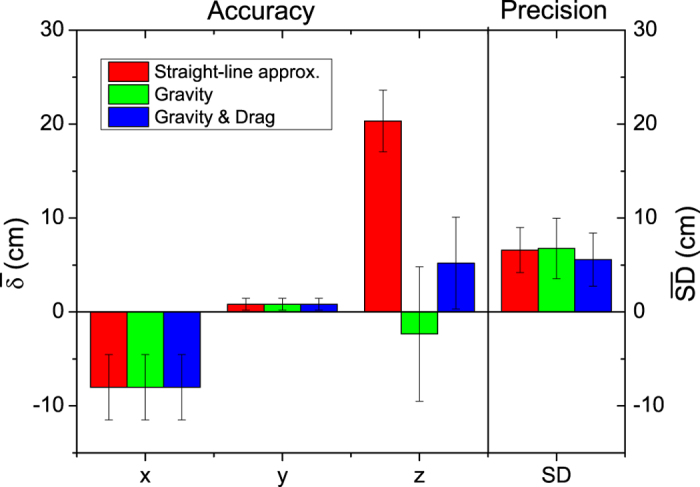
The accuracy and precision of the analysis of eight impact patterns. The mean deviation from true origin for the x, y and z coordinates and the standard deviation for the straight-line approximation (red), the method including gravity (green), and the method including both gravity and drag (blue). The bars represent the mean deviation measured for eight (n = 8) analyses performed. The error bars represent the variance of 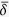 and 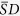.

We make a distinction between the Point of Origin (*PO*) and the Region of Origin (*RO*). The *PO* is the exact location given in x, y and z coordinates (*PO*(*x*, *y*, *z*)), which we calculated the pattern to be originated from. The *RO* is defined as twice the standard deviation (*SD*) of the shortest distance between the PO and each trajectory (see supplementary materials). In Fig. 3 an example is shown of the analysis of a typical impact pattern consisting of the straight-line approximation (red) and our method including gravity (green). The blue dot represents the location of the true origin. The spheres represent the *RO* and the black dot in the middle of the spheres represents the *PO*. For this specific pattern 31 stains were analyzed (red dots). The true origin of all patterns was at a height of 63.7 cm, (true origin *PO*_*origin*_(*x*, *y*, *z*) = (100,150, 63.7) in cm). It is clear that the straight-line approximation overestimates the point of origin height, *z*_*PO*_ = 91.1 cm, which is an overestimation of 27.4 cm. With our method we determine the height to be 58.5 cm, which actually differs only by −5.2 cm, which is almost one order of magnitude more accurate. The standard deviation caused by measurement errors (width, length and volume measurements) is somewhat larger for our method than for the straight-line approximation. Nevertheless, the true origin falls within the standard deviation of our method, which is not the case for the straight-line approximation. Next, we analyzed eight impact patterns with the straight-line approximation, our method including gravity, and our method including both gravity and drag. The precision and accuracy of all the results of the three methods are compared to one another (Fig. 4).

### Accuracy

The accuracy of our results can be defined as the difference between the location of the origin where we think the blood came from and the origin where it actually came from, i.e., the PO that we measure and the true origin for the x, y and z coordinates separately. Accordingly we determined the mean deviation 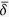 between PO and true origin for the x, y and z coordinates for these impact patterns:




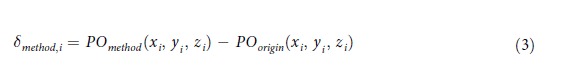







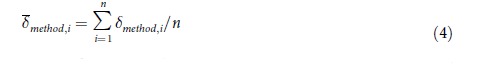




where *i* is the index number of the patterns and *PO*_*method*_ is the point of origin calculated by means of the various methods. In case of perfect accuracy, both *PO*_*method*_ and *PO*_*origin*_ are equal and accordingly 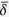 is zero. Thus an *increasing* 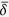 corresponds to a *decreasing* accuracy. Comparing the results from the straight-line approximation with our method including gravity (Fig. 4), it is clear that the x and y deviations are the same, as they should be, because they are calculated identically. In contrast, for the z coordinate the mean deviation is reduced dramatically, from roughly 20 cm for the straight-line approximation to roughly minus 2 cm for the gravity included method, which is an improvement of one order of magnitude, thus increasing accuracy. Even though the variance (error bar Fig. 4) of 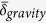 is larger than that of 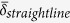, it still falls within the 20 cm mean deviation from the straight-line approximation. The method including both gravity and drag has a mean deviation of approximately 5 cm, which is higher than the gravity method, as expected (see discussion). In contrast, the variance of 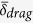 in Fig. 4 is much smaller than the variance of 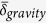. It is clear that the accuracy is highest for the method only including gravity. In addition, the straight-line approximation has the largest systematic error.

### Precision

The precision of our results can be defined as the spread of the trajectories in space at the point of origin. Accordingly, the size of the *RO* gives us information about the precision of our analysis with the various methods. Therefore, we determined the average size of the region of origin:




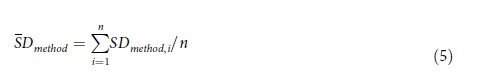




where *SD*_*method*,*i*_ is the standard deviation of the used method for one specific pattern (see supplementary methods). In case of perfect precision, all trajectories come together in one single point and the average standard deviation equals zero. Accordingly, an *increase* in 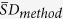 corresponds to a *decrease* in precision. Figure 4 shows the precision 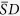, i.e., the average size of the red and green spheres from Fig. 3. It is clear that the standard deviation for our gravity method is slightly higher than for the straight-line approximation. In contrast, the standard deviation for the drag included method is smaller than both the straight-line and gravity method. Accordingly, including both gravity and drag gives the highest precision for the trajectory calculation.

### Maximum deviation

**Figure 5:**
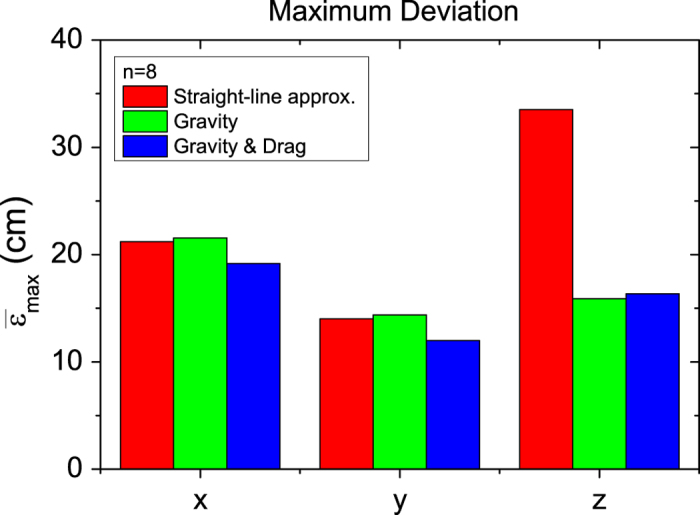
maximum deviation determined by means of the deviation from the true origin plus two times the standard deviation for the various methods used.

We determined the average maximum deviation 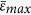 based on the different methods with a 95% confidence level (Fig. 5), by means of:




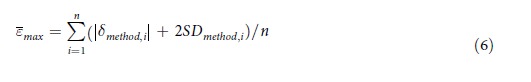




For the straight-line approximation we can have a deviation as much as 32 cm which is in accordance with the results of  De Bruin *et al.*^4^ who found similar results. Even though the straight-line approximation has a high precision, the accuracy is low because gravity and drag are neglected. For the gravity method, this maximum deviation diminishes to roughly 15 cm. In contrast to the straight-line approximation, the gravity included method has a very high accuracy and a high precision. Finally, 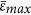 slightly increases for our drag method (roughly 16 cm), because the accuracy is lower even though the precision is higher. These results show that including gravity and drag, the origin can be determined at least two times more accurately than with the straight-line approximation, having a 95% confidence level.

## Discussion

The results reported in this paper show that we are able to determine the volume of small (≈1 *μ*l) bloodstains to determine the impact velocity of those stains and accordingly, the position where they came from. In addition, with our method we estimate the *PO* much more accurately than the straight-line approximation. As expected, the height estimation for the gravity method is on average below the true origin, because drag is not considered. Due to drag, the velocity decreases as the droplet flies through the air. But when tracing back the trajectory from the stain to its origin, time is inverted and the velocity increases due to drag. Accordingly, the drag included trajectories will always be between the straight line trajectory and the gravity trajectory, which explains why the drag included model has a higher, positive mean deviation compared to the gravity included model.

On the other hand, the droplets do not truly originate from a single point as, during an impact, the blood is first extruded into liquid sheets and ligaments, which in turn break up into droplets (see supplementary video). Only after this do the droplets follow the paths described by the gravitational and drag model. As the sheets and ligaments are distributed over a region of space, no method will find all trajectories crossing at a single point, i.e., 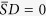, no matter how accurate the measurement of the stain dimensions or how physically correct the trajectory model. In addition, due to the physical limitations of our setup, the breakup occurs around and above the actual point of impact of the weapon. Thus the height estimation will always be slightly overestimated, i.e., 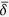 for z will always be nonzero and positive. The results from our model including both gravity and drag reflect this overestimation well. Finally, the equations of motion of the blood droplets become more non-linear if air drag is included, since drag depends on the speed of the droplet itself. It is entirely possible that this leads to errors that propagate in the reconstruction of the trajectory, leading in turn to the increased deviation.

An elaborate study^21^ based on computer modeling showed that typical indoor air currents can have a significant effect on the trajectories of airborne droplets. Standards on indoor air conditions^22^ limit the maximum permissible air velocity in dwellings to 0.3 m/s. Based on indoor air current of 0.5 m/s the recorded flight path deviations were less than 2 cm for a droplet of 5 mm in diameter (deposition height 1.4 m) which could increase up to 2–3 m for a droplet of 0.1 mm in diameter^21^. Based on these findings, air currents can influence bloodstain patterns and thus the determined location where they originate from, which is worthy of further investigation.

The standard deviation decreases (increase of precision) when gravity (and drag) are taken into account for the trajectory reconstruction, because the trectories lie closer to one other. This increase of precision is surprising as more variables (volume and impact velocity) are introduced to the calculation, increasing the number of random errors. However, by implementing gravity and drag, the trajectories follow a more realistic path and thus increasing precision. The precision of our method might even be increased further by removing outliers from our data set^23^, however, this falls outside the scope of this investigation.

Various studies have attempted to increase the accuracy of directional analysis^3^,^5^,^7^,^24^,^25^, but to our knowledge, none of these methods are used on the crime scene. Nevertheless, there is a high demand for improving the scientific methods used within BPA on the crime scene^26^,^27^. Our new method enables investigators to distinguish between a sitting and standing position or might even connect specific patterns to wounds created during the bloodletting event. Accordingly, the investigator will be able to reconstruct the events of the crime in more detail.

This method can be used on the crime scene as the 3D scanner can easily be transported. In addition, our method is non-invasive and contact-less, a prerequisite for using it in forensic applications, considering contamination. These attributes make the scanner suitable to use on the crime scene. With only one additional measurement, namely the 3D scan, we create many possibilities concerning the reconstruction of the trajectories. In addition, the analysis of the bloodstains does not increase in complexity when compared to directional analysis.

On a crime scene it is not unlikely that the majority of bloodstains found are downward directed. With this method it might be possible to take downward directed bloodstains into account. These kinds of bloodstains are now excluded in the analysis as the height estimation becomes very inaccurate. However, by increasing the origin distance from the wall, more downward directed stains will be present.

Finally, we apply our volume and velocity measurements only on impact bloodstain patterns. However, this method might also be used for analyzing other kinds of bloodstain patterns. For example, the drip trail pattern (stains resulting from droplets, created solely by gravity) could be investigated to determine the average height of deposition^28^. Moreover, cast-off patterns (patterns created due to the rapid movement of a bloody object) could be investigated to determine the swing radius and maybe even the radial velocity of the object^29^.

## Conclusion

By means of these proof-of-principle experiments, we show that with our method we arrive at a much more accurate and precise determination of t.he point and region of origin when performing the analysis for stains that are selected by BPA experts. It is evident that the accuracy can be further improved by taking also the downward directed stains into account which are usually discarded on the crimes scene, but this is subject of future study. When using the straight-line approximation, only upward directed bloodstain can be considered for analysis as downward directed bloodstains could have been influenced by gravity. If so, this introduces an unacceptably large margin of error for the downward directed stains, which could constitute the majority of stains found at a crime scene. We anticipate this study to be of considerable importance for investigators who use BPA to reconstruct the events that took place on crime scenes. The improved accuracy will allow them, for instance, to better determine the position of the victim or it might be possible to connect bloodstain patterns to specific wounds on the body, which differ in height.

## Additional Information

**How to cite this article**: Laan, N. *et al.* Bloodstain Pattern Analysis: implementation of a fluid dynamic model for position determination of victims. *Sci. Rep.* **5**, 11461; doi: 10.1038/srep11461 (2015).

## Supplementary Material

Supplementary Video S1

Supplementary Information

## Figures and Tables

**Figure 1:**
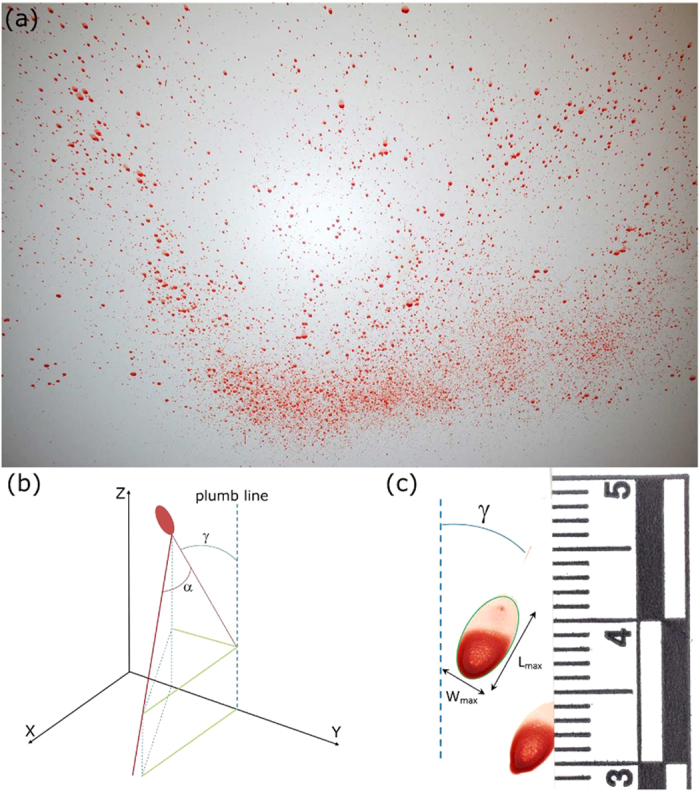
Example of a bloodstain impact pattern with a detailed photograph of a single bloodstain. (**a**) Impact pattern created by means of a hammer on spring released into a volume of blood. (**b**) Schematic representation of the directional angle *γ* and the impact angle *α* of a single bloodstain (red ellipse). (**c**) A single elliptical bloodstain of which the tail shows the direction of travel.
