# Supplementary material for: Bloodstain Pattern Analysis: implementation of a fluid dynamic model for position determination of victims
Source: Sci Rep. 2015 Jun 22;5:11461. doi: 10.1038/srep11461 (PMC4476491; doi:10.1038/srep11461)
Supplement: Supplementary Information [file srep11461-s2.pdf]

Supplementary Information to:

Bloodstain Pattern Analysis: implementation of a fluid dynamic model for position determination of victims.

Nick Laan[1,2]*, Karla G. de Bruin[2], Denise Slenter[1], Julie Wilhelm[1,3], Mark Jermy[3] and Daniel Bonn[1]

[1]WZI, IoP, Science Park 904, University of Amsterdam, Amsterdam, Netherlands

[2]Laan van Ypenburg 6, Netherlands Forensic Institute, The Hague, Netherlands

[3] Private Bag 4800, Christchurch 8140, University of Canterbury, New Zealand

Corresponding author's email address: n.laan@uva.nl

S1. Physical properties of blood

Table S1 | Physical properties of blood

	**Hct** %	**Density** kg/m^3	**Surface tension** mN/m	**Viscosity** mPa·s	**At temp.** °C
**Blood**	41%	1055±3	60±2	4.8	22
**Blood (dried)**	-	1274±3	-	-	-

We used human blood which was obtained with the same procedure as[8]. For each blood sample collected, the hematocrit (Hct) value (the percentile amount of red blood cells in blood) was determined by means of a capillary centrifuge (Haematokrit 210, Hittich Zentrifugen, germany). The density of liquid blood (ρ_{blood}) was determined by means of weighing a (empty and blood filled) volumetric flask of 10 ml (Hirschmann, Germany). In addition, the density of dried blood was measured using a gas expansion pycnometer (Micromeritics Multivolume Pycnometer 1305, USA). The viscosity of blood was determined by using a rheometer (MCR 302, Anton Paar, Austria) with a cone and plate geometry, using a shear rate sweep, (1 s^{-1} < $\dot{\gamma}$ < 1000 s^{-1}). Blood is shear thinning but viscosity reaches a plateau value for high shear rates[9], see Figure S1. The high shear rate viscosity η_∞ was determined by fitting the phenomenological equation:

$$\eta(\dot{\gamma}) = \eta_\infty + k \cdot \dot{\gamma}^{n-1} \qquad \text{(S1)}$$

where k [Pa·s^n] and n are fit parameters. The viscosity of blood was determined for both room temperature (22°C) and body temperature (38°C). The surface tension σ was determined using the pendant drop method (Easydrop, Krüss, GmbH, Germany).

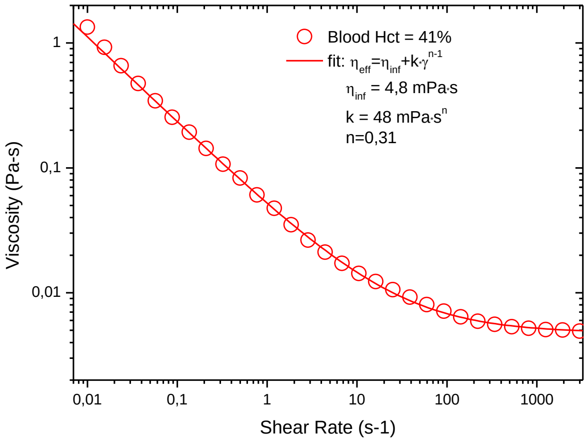

Figure S1 | Viscosity as a function of shear rate, $\dot{\gamma}$, of blood at 22 °C (circles). For high shear rates, the viscosity reaches a constant, which was determined by means of fitting $\eta(\dot{\gamma}) = \eta_\infty + k \cdot \dot{\gamma}^{n-1}$ to the data points (red line), from which we obtain a high shear rate viscosity of η_∞ = 4.8 mPa·s; k = 0.048 mPa·s^n and n = 0.31 are fitting constants.

To determine the hematocrit dependency for the drying ratio, blood with varying hematocrit values were required. To do so, multiple blood samples (4 ml) were obtained from one volunteer. The samples were centrifuged to separate the plasma from the red blood cells. Accordingly, plasma was drawn from one sample, thus effectively increasing the Hct value and added to another sample, decreasing its Hct value. In this manner, nine blood samples with Hct values between 23% and 54% were created.

S2. Volume estimation

To determine the impact velocity of the droplet we require the original droplet volume[5-7,30]. After a bloodstain has dried, it leaves a residue, consisting of dried red blood cells, the amount of which is dependent on the hematocrit value[8]. It is possible to deduce the original volume of a bloodstain by measuring the volume of the dried stain and divide it by the volume ratio of dried and fresh blood. To measure the volume we use a 3D surface scanner.

Material and methods

The AreaScan3D (VRMagic, Germany) records the deformation of a regular projected light pattern. The scanner consists of a camera which is positioned perpendicular to the surface, at a distance of 25 cm. Next to the camera there is a projector, which is inclined with respect to the surface under an angle of approximately 80°, which projects several light patterns onto the surface. Due to the inclination and height variations of the surface, the camera records distorted lines from which height information can be obtained. The scanner has a lateral range of 1.8 by 1.2 cm (748x480 pixels) with step sizes of 24.064 μm/pixel and 25 μm/pixel, respectively. The axial range is one centimeter, with a height resolution in the order of a micrometer. Each scan is in the form of a point cloud with x, y and z coordinates. By means of a software program (written in Java), we select the object (bloodstain) based on the height difference of the object relative to the surface, from which we directly determined the volume. Figure S2 shows a single bloodstain from an impact pattern together with the 3D scan (Figure S2b, intensity graph). In addition, from the cross-section of the bloodstain (Figure S2c) it is possible to see the difference between the irregular surface and the bloodstain.

Surface irregularities are taken into account by determining the mean in height deviations over a large area of the surface, without the object. The total volume V_{tot} is determined by selecting the object and accumulating the height differences with respect to the surface. The selected area is multiplied with the mean of the surface irregularities which results in $V_{surface}$. The volume of the object V_{netto} is determined by subtracting $V_{surface}$ from V_{tot}.

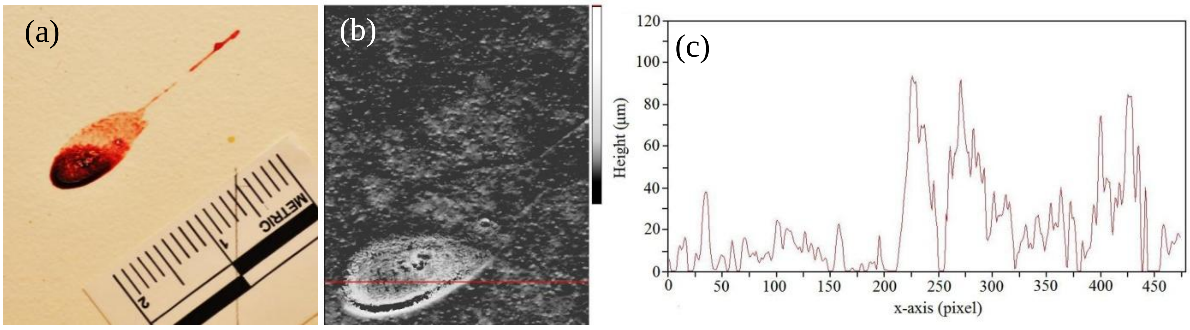

Figure S2 | A single bloodstain from an impact pattern. (a) a photograph of a bloodstain including a scale bar. (b) The intensity graph of the bloodstain obtained from the AreaScan3D. (c) The cross-section of the bloodstain corresponding to the red curve from (b), where the height variation is plotted as a function of the lateral axis (x-direction).

To validate the volume measurements using the AreaScan3D, we created small aluminum cylinders with predefined volumes, ranging from 1 to 140 µl, as a reference. The volume obtained from these scans we compared to the volume obtained from the weight and density (2701 kg/m^3)[31] of the aluminum cylinders. In addition, we created bloodstains of different sizes (5 to 50 µl) by means of a pipette, and bloodstains (7 µl) by means of a needle and deposited from several heights (10 to 100 cm). Each bloodstain was created on a separate surface and weighed (immediately after deposition and after drying) to determine the volume by means of the density of blood (table S1), to compare this to the volume obtained from the AreaScan3D. After creation of a single bloodstain or a bloodstain pattern, the blood was dried for a minimum of three hours. After this time period the blood was completely dried after which a 3D scan was made of the bloodstains.

Results

There is a very high correlation between the volume measured by means of the AreaScan3D and the volume obtained from the weight/density measurement, of the aluminum cylinders (Figure S3). By fitting a line to the data points ($y = 0.979 \cdot x$), we see there is only a small variation of 2.1% between the measured volumes. It is clear that the AreaScan3D has a very high accuracy for determining volumes.

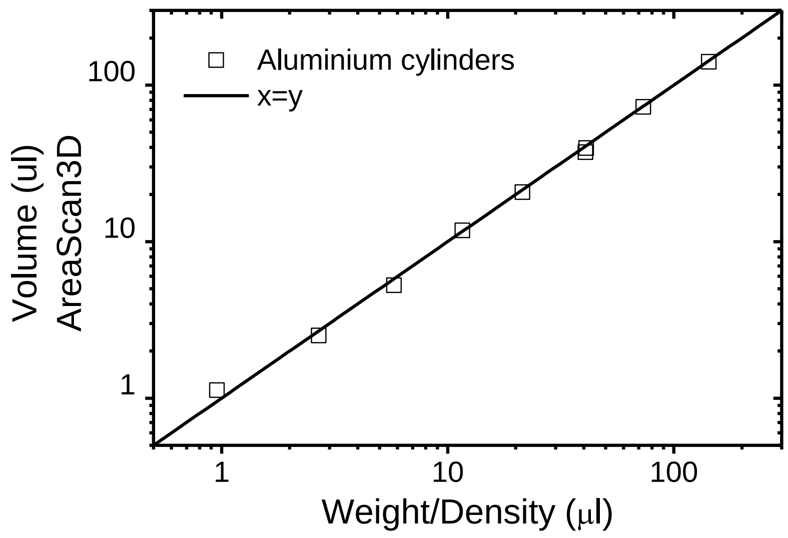

Figure S3 | the volume of the aluminum cylinders measured by means of the AreaScan3D as a function of the volume obtained by means of the weight and density.

However, the question remains if we are able to determine the volume of bloodstains just as accurately. By plotting the dried weight of the bloodstains as a function of the relative dried volume (Figure S4) we are able to determine the density of the dried bloodstains (1546 kg/m^3) as measured by our system. However, the actual density of dried blood is lower, namely 1274 kg/m^3. The volume we measure with the AreaScan3D is thus too low. However, this density deviation is a systematic error, which we can account for by means of a calibration curve (Figure 2, main article).

The systematic error of the AreaScan3D is caused by light absorption of the blood. The AreaScan3D projects white light onto the sample and captures the diffuse reflectance with a camera. Blood itself is highly absorbent in the white light range[32]. Accordingly, less light is reflected back into the camera and loss of information occurs, which causes the measured systematic error for which we are able to correct with our calibration curve.

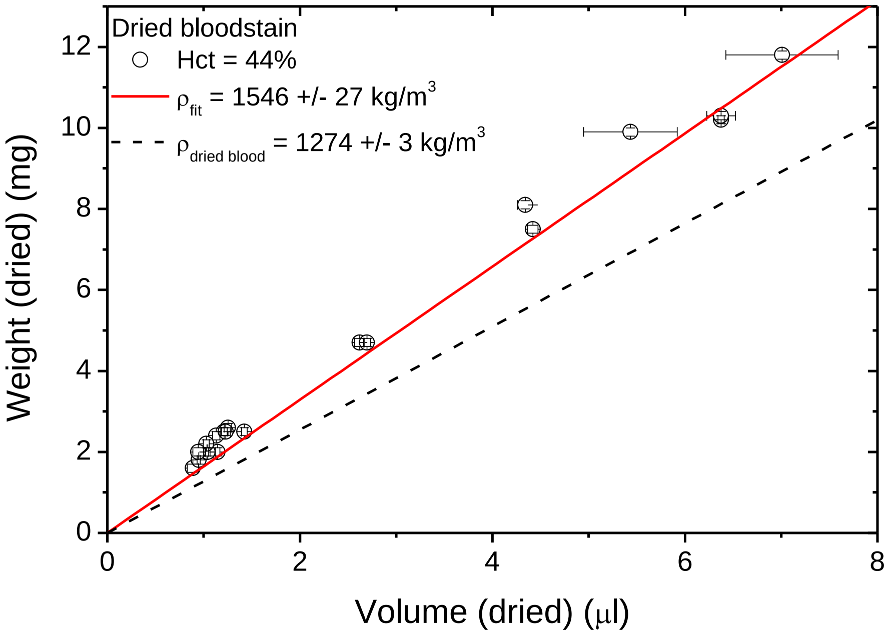

Figure S4 | volume measurements of bloodstains. Weight of dried bloodstains plotted as a function of the volume of dried bloodstains. From the fit to the data points we obtain the density of blood.

S3. Trajectory reconstruction

We made eight bloodstain impact patterns at a distance of one meter from the wall at a height of 63.7 cm, using a hammer on a spring setup, described in de Bruin *et al.*[4] and using the same measurement procedure to determine the impact angle and location of each bloodstain. For each bloodstain pattern we took ~2 mL of blood which was heated to 37° to simulate a real blood shedding event. After drying a certified bloodstain pattern analyst chose any number of available bloodstains. After selection, a picture of each chosen stain including a two centimeter reference length (Figure S1a) was taken (Nikon D5200, 4mm macro lens) at a fixed distance from the wall, to determine the width, length, directional angle γ and impact angle α of the bloodstains. In addition, each selected stain was scanned with the AreaScan3D to determine the volume. The locations of the stains were determined by measuring the distance from the floor and a side wall with a digital distometer (Disto X310, Leica). All measurements were performed in the same space on the same walls in the Netherlands Forensic Institute.

The PO and RO were determined by means of a straight line approximation, by means of our method including gravity, and by both including gravity and drag. With the gravity method the curved trajectories were determined by solving the equations of motion analytically for a ballistic object taking only gravity into account.

$$m\vec{a} = \vec{F_g} \quad \text{(S2)}$$

$\vec{F_g} = m\vec{g}$ is the gravitational force with m the mass of the droplet and $\vec{g}$ the gravitational acceleration. Eq. 2 can be solved for the motion in the x, y and z plane separately:

$$x(t) = x_0 + v_x t \quad \text{with} \quad v_x = v_0 \sin\alpha \quad \text{(S3)}$$

$$y(t) = y_0 + v_y t \quad \text{with} \quad v_y = v_0 \sin\gamma\cos\alpha \quad \text{(S4)}$$

$$z(t) = z_0 + v_z t - \frac{1}{2} g\, t^2 \quad \text{with} \quad v_z = v_0 \cos\gamma\cos\alpha \quad \text{(S5)}$$

with impact angle α, the directional angle γ and the impact velocity v_0.

To take drag into account the equations of motion have to be solved numerically as they cannot be solved analytically. The trajectories of the blood droplets were modeled as described in Kabaliuk *et al*.[19] using a Lagrangian approach for Newton's second law of motion:

$$m\vec{a} = \vec{F_g} + \vec{F_b} + \vec{F_D} \tag{S6}$$

where $\vec{F_b} = \frac{\pi}{6} {D_0}^3 \rho_{air} \vec{g}$ is the buoyancy force with ρ_{air} the density of air, $\vec{F_D} = -\frac{1}{2} C_D A \rho_{air} \vec{v_{rel}}^2$ is the drag force with C_D the drag force coefficient, *A* the projected area of the droplet and $\vec{v_{rel}}$ the relative velocity of the droplet during flight. Eq. 6 can be rewritten for the motion in the x, y and z plane:

$$a_x = \frac{dv_x}{dt} = \frac{1}{2m} C_D A \rho_{air} \frac{v_{relx}}{|\vec{v_{rel}}|} \tag{S7}$$

$$a_y = \frac{dv_y}{dt} = \frac{1}{2m} C_D A \rho_{air} \frac{v_{rely}}{|\vec{v_{rel}}|} \tag{S8}$$

$$a_z = \frac{dv_z}{dt} = \frac{1}{2m} C_D A \rho_{air} \frac{v_{relz}}{|\vec{v_{rel}}|} - g \frac{\rho - \rho_{air}}{\rho} \tag{S9}$$

with v_{relx}, v_{rely} and v_{relz} the relative velocities in the x, y and z directions, respectively. These equations are solved numerically for discrete time steps using a fourth order Runge-Kutta algorithm. In addition, deformation of the droplet shape during flight can be taken into account with the Taylor Analogy Breakup model (TAB)[33]. For this study, the blood drops were modeled as solid spheres (no deformation and a constant drag coefficient equal to 0.5). This model was found to be the most suited for this practical situation.

S4. Point of origin and Region of origin determination

Point of Origin

The intersections between the trajectories were determined within the x-y plane (Figure S5)[2]. By taking the arithmetic mean of the intersecting points ($\mathrm{O}(\mathrm{x}_i, \mathrm{y}_i) = \sum_k \mathrm{O}_k(\mathrm{x}_k, \mathrm{y}_k)/\mathrm{k}$, where *k* is

the index number for the intersecting points and *i* the index number for the pattern), the x and y coordinates were determined of the PO, which are the same for each method (the projection of the trajectories onto the x-y plane does not change due to gravity or drag). For each trajectory, the z_i coordinate was determined based on the mean of the z_k (the height of the trajectory at $O(x_i,y_i)$);

$$\mathrm{PO}_{method}(x_i, y_i, z_i) = \sum_{\mathrm{k}} \mathrm{O_k}(\mathrm{x}_i, \mathrm{y}_i, \mathrm{z}_k)/\mathrm{k}) \quad \text{(S10)}$$

where negative values of z_k were assumed zero due to the physical limitation of the floor (z = 0). The obtained $PO(x_i, y_i, z_i)$ is assumed to be the point of origin.

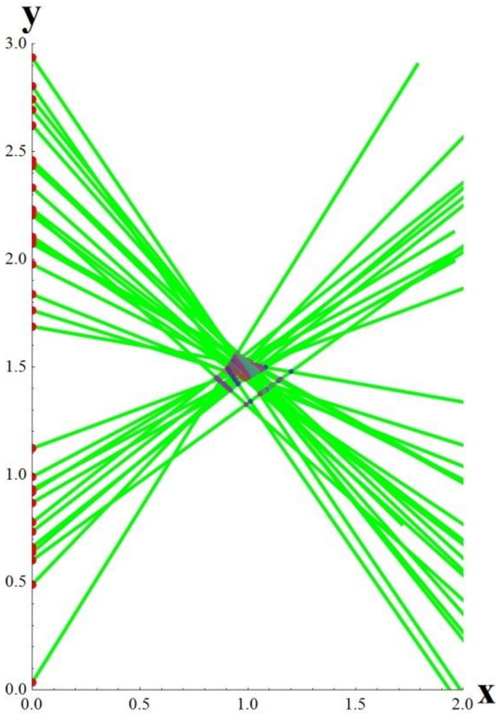

Figure S5 | the trajectories of the blood droplets in the x-y plane. The wall containing the bloodstain is on the left (x=0). The points depict the intersections between the upward and downward directed trajectories within this x-y plane, distinguished by positive and negative γ values.

Region of origin

The region of origin is determined by taking the standard deviation into account, which was determined from the smallest distance between the point of origin and each trajectory. The standard deviation given in Eq. 4 (main article) is determined by means of the following

method. First of all, the PO is determined by means of Eq. S10. Next we define the distance between the PO and each trajectory (index p) as a function of time.

$$S_p(t) = \sqrt{(\mathrm{PO}(x_i) - x_p(t))^2 + (\mathrm{PO}(y_i) - y_p(t))^2 + (\mathrm{PO}(z_i) - z_p(t))^2} \qquad \text{S11}$$

here $x_p(t)$, $y_p(t)$ and $z_p(t)$ are the time dependent trajectories of the droplets given by equations S3, S4 and S5, respectively. To determine the shortest distance (for the straight-line method and gravity included method) we solve the time derivative for t of Eq. S11 equaling zero, yielding a t_{min}, which is filled into Eq. S11 for each trajectory. For the method including both gravity and drag it is not possible to determine the derivative of Eq. S11 because the trajectories are numerically solved. Thus, Eq. S11 is directly numerically calculated by determining $S_p(t)$ for each time step (Δt=0.1 ms). Accordingly, the shortest distance of each trajectory is determined. Next, the standard deviation of the minimum distances is calculated for each pattern.

$$SD = \sqrt{\frac{1}{L}\sum_{p=1}^{L}(S_p(t_{min}) - \mu)^2} \quad \text{where} \quad \mu = \frac{1}{L}\sum_{p=1}^{L} S_p(t_{min}) \qquad \text{S12}$$

where L is the number of trajectories.

S5. Supplementary video

The supplementary video shows a high speed recording of a hammer striking a volume of liquid blood (~2 ml). The video was recorded with 3000 frames per second, using a high speed camera (Photron). As the hammer hits the blood, sheets and ligaments of blood disperse away from the impact area, which in turn, breaks up in droplets several centimeters away from the impact.

2 Carter, A.L., The directional analysis of bloodstain Patterns - Theory and Experimental Validation. *Can. Soc. Forens. Sci. J* **34**, 173-189 (2001).

4 de Bruin, K.G., Stoel, R.D., & Limborgh, J.C.M., Improving the Point of Origin Determination in Bloodstain Pattern Analysis. *J. Forensic Sci.* **56**, 1476-1482 (2011).

5 Hulse-Smith, L., Mehdizadeh, N.Z., & Chandra, S., Deducing drop size and impact velocity from circular bloodstains. *J. Forensic Sci.* **50**, 54-63 (2005).

6 Hulse-Smith, L. & Illes, M., A Blind Trial Evaluation of a Crime Scene Methodology for Deducing Impact Velocity and Droplet Size from Circular Bloodstains. *J. Forensic Sci.* **52**, 65-69 (2007).
7 Knock, C. & Davison, M., Predicting the Position of the Source of Blood Stains for Angled Impacts. *J. Forensic Sci.* **52**, 1044-1049 (2007).
8 Laan, N., Bremmer, R.H., Aalders, M.C.G., & de Bruin, K.G., Volume Determination of Fresh and Dried Bloodstains by Means of Optical Coherence Tomography. *J. Forensic Sci.* **59**, 34-41 (2014).
9 Laan, N., de Bruin, K.G., Bartolo, D., Josserand, C., & Bonn, D., Maximum Diameter of Impacting Liquid Droplets. *Phys. Rev. Appl.* **2**, 044018 (2014).
19 Kabaliuk, N., Jermy, M.C., Williams, E., Laber, T.L., & Taylor, M.C., Experimental validation of a numerical model for predicting the trajectory of blood drops in typical crime scene conditions, including droplet deformation and breakup, with a study of the effect of indoor air currents and wind on typical spatter drop trajectories. *Forensic Sci. Int.* **245**, 107-120 (2014).
30 Lee, H.C., Estimation of original volume of bloodstains. *Identification News* (1986).
31 Haynes, W.M., *CRC Handbook of Chemistry and Physics.* (CRC Press, Boca Raton, FL, 2010).
32 Bremmer, R.H., Kanick, S.C., Laan, N., Amelink, A., van Leeuwen, T.G., Aalders. M.C.G., Non-contact spectroscopic determination of large blood volume fractions in turbid media. *Biomed. Opt. Express*. **2**. 396-407 (2011)
33 O'Rourke, P.J. & Amsden, A.A., The TAB method for numerical calculation of spray droplet breakup. SAE Technical Paper (1987).
